# Exploring the salt–cocrystal continuum with solid-state NMR using natural-abundance samples: implications for crystal engineering

**DOI:** 10.1107/S205225251700687X

**Published:** 2017-06-05

**Authors:** Lalit Rajput, Manas Banik, Jayasubba Reddy Yarava, Sumy Joseph, Manoj Kumar Pandey, Yusuke Nishiyama, Gautam R. Desiraju

**Affiliations:** a Solid State and Structural Chemistry Unit, Indian Institute of Science, Bangalore, 560 012, India; b RIKEN CLST–JEOL Collaboration Center, RIKEN, Yokohama, Kanagawa 230-0045, Japan; c JEOL RESONANCE Inc., Musashino, Akishima, Tokyo 196-8558, Japan; d Department of Chemistry, Indian Institute of Technology Ropar, Rupnagar, India

**Keywords:** salt, cocrystal, continuum, natural abundance, solid-state NMR, X-ray diffraction

## Abstract

There has been significant recent interest in differentiating multicomponent solid forms, such as salts and cocrystals, and, where appropriate, in determining the position of the proton in the *X*—H⋯*A*—*Y* *X* ^−^⋯H—*A* ^+^—*Y* continuum in these systems, owing to the direct relationship of this property to the clinical, regulatory and legal requirements for an active pharmaceutical ingredient (API). In the present study, solid forms of simple cocrystals/salts were investigated by high-field (700 MHz) solid-state NMR (ssNMR) using samples with naturally abundant ^15^N nuclei. Four model compounds in a series of prototypical salt/cocrystal/continuum systems exhibiting {PyN⋯H—O—}/{PyN^+^—H⋯O^−^} hydrogen bonds (Py is pyridine) were selected and prepared. The crystal structures were determined at both low and room temperature using X-ray diffraction. The H-atom positions were determined by measuring the ^15^N—^1^H distances through ^15^N-^1^H dipolar interactions using two-dimensional inversely proton-detected cross polarization with variable contact-time (invCP-VC) ^1^H→^15^N→^1^H experiments at ultrafast (ν_R_ ≥ 60–70 kHz) magic angle spinning (MAS) frequency. It is observed that this method is sensitive enough to determine the proton position even in a continuum where an ambiguity of terminology for the solid form often arises. This work, while carried out on simple systems, has implications in the pharmaceutical industry where the salt/cocrystal/continuum condition of APIs is considered seriously.

## Abstract 2

The salt–cocrystal continuum is a well known phenomenon in crystal engineering and has been studied here in several multicomponent solids with solid-state NMR (700 MHz) using ^15^N-^1^H heteronuclear dipolar coupling. The measurement is made at ultrafast (60–70 kHz) magic angle spinning (MAS) frequency. The experiment is sensitive enough to determine the proton position even in a continuum situation and can be performed on minimal amounts of microcrystalline or even amorphous solids with natural-abundance ^15^N samples. Such a measurement gives reliable values of N—H distances and is therefore a direct indication of the position of the proton in the salt–cocrystal continuum. The crystal structures of the relevant solids have also been determined at a high level of accuracy and the results of the X-ray and NMR experiments are compared.

## 1. Introduction   

Detection of the H-atom position in an *X*—H⋯*A*—*Y* hydrogen bond is a matter of fundamental and practical importance (Jeffrey, 1997 ▸; Desiraju & Steiner, 1999 ▸). Atomic positions derived for an H atom from an X-ray analysis approximates the centroid of the electron density. Positions derived from neutron diffraction correspond to the nuclei of the atoms. Neutron-derived H-atom positions and the corresponding hydrogen-bond metrics are more accurate but this does not necessarily mean that they are chemically the most meaningful (Aakeröy & Seddon, 1993 ▸; Cotton & Luck, 1989 ▸). An *X*—H⋯*A*—*Y* hydrogen bond may also be considered as an extreme of a proton-transfer reaction where the other extreme is *X* ^−^⋯H—*A* ^+^—*Y*. Situations are known in which this proton-transfer reaction is mediated by a change in tem­per­ature and where the H-atom position varies smoothly between the *X* and *A* atoms (Steiner *et al.*, 2001 ▸; Parkin *et al.*, 2004 ▸; Wilson & Goeta, 2004 ▸; Grobelny *et al.*, 2011 ▸).

**Figure 1:**
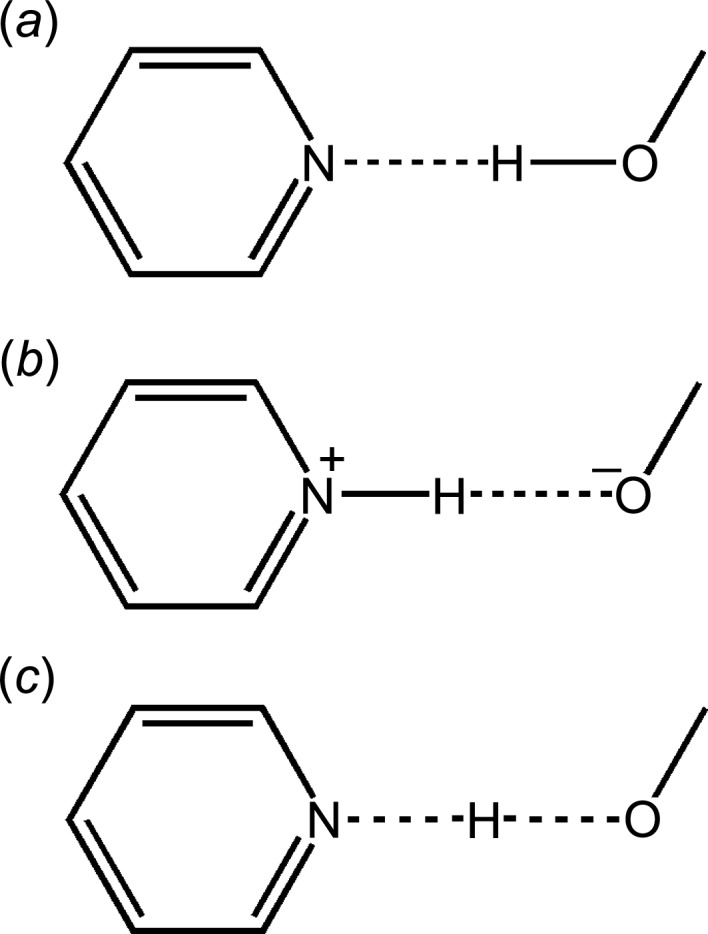
Schematic presentation of (*a*) a cocrystal, (*b*) a salt and (*c*) a continuum (where the H-atom position is shared between the two heavy atoms) in a typical O···H···N interaction.

In the pharmaceutical industry, there is considerable interest in making multicomponent molecular crystals of drugs or active pharmaceutical ingredients (APIs) in order to achieve better physical, chemical or pharmacological properties (Almarsson & Zaworotko, 2004 ▸; Vishweshwar *et al.*, 2006 ▸; Wouters & Quéré, 2012 ▸). Generally, these crystals involve the formation of a hydrogen bond between the drug molecule, which is usually basic, and another compound, referred to often as a coformer, which is usually acidic. The binary crystal therefore often contains a hydrogen bond of the type (Drug)⋯H—(Coformer) and if the two molecular species are not ionized, the substance is called a ‘cocrystal’. The definition of this term ‘cocrystal’ is still contentious (Desiraju, 2003 ▸; Dunitz, 2003 ▸; Bond, 2007 ▸; Childs *et al.*, 2007 ▸; Aakeröy & Salmon, 2005 ▸; Aitipamula *et al.*, 2012 ▸) and is in some aspects incomplete or inadequate. In any event, if the proton-transfer reaction across the hydrogen bond is complete, the multicomponent crystal that is obtained is of the form (Drug)^+^—H⋯(Coformer)^−^ and is called a ‘salt’, whereas the intermediate state of affairs is termed a ‘continuum’ (Fig. 1 ▸).

For the patent protection of new multicomponent solid forms of an API, the substance to be patented must be characterized as denoted by the specifications, *i.e.* whether it is a salt or cocrystal. For example, some APIs were found to exhibit the tendency to transform from one drug form to another due to external forces, such as light, heat, pressure and mechanical grinding (Ikni *et al.*, 2014 ▸; Pirttimäki *et al.*, 1993 ▸; Shakhtshneider & Boldyrev, 1993 ▸; Otsuka *et al.*, 1994 ▸). Therefore, a proper study of the new solid form is of the utmost importance as it is directly related to both patient safety and clinical efficacy. If this issue remains unresolved, the exploration of pharmaceutical solids becomes restricted and the competitive advantage of drug development to launch the product is lost. There are important regulatory and legal implications as to whether or not the marketed form of a drug is the ‘cocrystal’ or ‘salt’ form. What is of relevance here is the so-called Δp*K*_a_ rule, which states that a salt is obtained if the p*K*_a_ difference between the drug and the coformer is greater than 3, while if the difference is less than 1, a cocrystal is obtained (Bhogala *et al.*, 2005 ▸; Cruz-Cabeza, 2012 ▸; Ramon *et al.*, 2014 ▸; Mukherjee & Desiraju, 2014 ▸; US–FDA, 2016 ▸). The intermediate region, *i.e.* 1 < Δp*K*_a_ < 3, contains cases where the H atom (proton) is unusually labile and wherein it can move between the drug and coformer species. This gives rise to the so-called ‘salt–cocrystal continuum’, a phenomenon that has been studied using a variety of crystallographic techniques (Aakeröy *et al.*, 2007 ▸; Childs *et al.*, 2007 ▸; Schmidtmann *et al.*, 2007 ▸; Hathwar *et al.*, 2010 ▸; Thomas *et al.*, 2010 ▸; da Silva *et al.*, 2013 ▸). Drug⋯coformer systems in the intermediate Δp*K*_a_ range are of special significance in regulatory and legal contexts. Over the last decade, crystal engineering has been used extensively to modify the physicochemical properties of APIs by making new solid forms (Desiraju, 2013 ▸; Duggirala *et al.*, 2016 ▸).

Solid-state NMR (ssNMR) methods have always been used in conjugation with diffraction methods, or alone, to determine H-atom positions in hydrogen bonds (Berglund & Vaughan, 1980 ▸; Rohlfing *et al.*, 1983 ▸; Jeffrey & Yeon, 1986 ▸; Wu *et al.*, 1998 ▸; Yazawa *et al.*, 2012 ▸; Miah *et al.*, 2013 ▸). ssNMR has also been used to ascertain the nature of the salt–cocrystal continuum in API systems (Stevens *et al.*, 2014 ▸). The use of ^15^N ssNMR has been documented previously (Li *et al.*, 2006 ▸). These applications rely on the dependence of chemical shift tensors on H-atom positions. It is obvious that ^1^H chemical shift tensors are very sensitive to H-atom positions because ^1^H NMR detects the H atoms directly. In addition, ^15^N chemical shift tensors are also affected by H-atom positions through changes in the electron distribution, thereby making it also sensitive to H-atom positions. These dependences are further corroborated by quantum chemical calculations. There are several cases where it is very difficult to obtain X-ray-quality single crystals, and powder X-ray methods are often inadequate to accurately determine H-atom positions. In addition, H-atom positions in X-ray diffraction measurements are systematically foreshortened and are at the limit of detection in any case. In such cases, ssNMR is an excellent complementary technique for determining H-atom positions. There is always the fundamental question of whether the X-ray-derived or the neutron-derived position is the more ‘correct’ or indeed if either of these positions is ‘correct’ at all. There are some studies that record differences between the H-atom positions determined by diffraction- and NMR-based methods (Roberts *et al.*, 1987 ▸; Lorente *et al.*, 2001 ▸). These disagreements arise partly from the uncertainty in the positions of the H atoms in *all* X-ray-diffraction-based methods on the one hand, and the fact that the determination of H-atom positions in ssNMR is based on an indirect measurement through chemical shift tensors on the other. Neutron diffraction analysis is very difficult to carry out, compared to X-ray diffraction and ssNMR, because large crystals are needed, which are often impossible to obtain, and also because one needs to collect data over an extended time period in a remote laboratory equipped with a neutron source. Noting that all three methods, *i.e.* X-ray diffraction, neutron diffraction and ssNMR, may give slightly different results for H-atom positions, and that none of these measurements can, strictly speaking, be used as benchmarks for each other, we embarked on the present study.

Besides the chemical shift tensor, ssNMR is able to measure internuclear N—H distances through ^15^N-^1^H dipolar interactions, as the magnitude of the dipolar interaction is inversely proportional to the cube of the internuclear distance. This potentially gives a straightforward solution to the ‘salt/cocrystal/continuum’ problem, while diffraction-based methods provide internuclear distances from the atomic positions themselves. However, the ^1^H—*X* distance measure­ments are not easy because of the presence of intense homonuclear ^1^H-^1^H dipolar interactions and a very low abundance of ^15^N (0.4%). While the former obscures the ^1^H-*X* dipolar interactions, the latter gives very limited sensitivity, making the measurements practically impossible. These difficulties require isotopic dilution of ^1^H with ^2^H and/or isotopic enrichment with ^15^N nuclei, thus limiting their common application. Moreover, the small ^15^N-^1^H dipolar interaction due to the small ^15^N gyromagnetic ratio (about 10% of ^1^H) complicates the problem further.

Recent progress in fast magic angle sample spinning (MAS) technology (Nishiyama, 2016 ▸) has paved a new way for determining ^1^H-*X* dipolar interactions (Paluch *et al.*, 2013 ▸, 2015 ▸; Park *et al.*, 2013 ▸; Zhang *et al.*, 2015 ▸; Nishiyama *et al.*, 2016 ▸). Ultrafast MAS > 60 kHz, which is exclusively achieved by a very tiny rotor with a diameter of less than 1.3 mm accompanied by a small sample volume, can overcome the above-mentioned difficulties by suppressing the ^1^H-^1^H dipolar interactions to facilitate ^1^H—*X* distance measurement. In addition, ultrafast MAS allows direct observation of the NMR signal through the ^1^H nucleus, which is much more sensitive than ^15^N because of its high gyromagnetic ratio. The use of high-field magnets, which are now commonly available to researchers, improves the sensitivity further. These combinations allow the observation of ^1^H-*X* dipolar interactions in natural-abundance samples with a limited sample volume. The one possible limitation of ^1^H observation in multicomponent systems is the poor resolution of the ^1^H chemical shift obtainable in ssNMR even at the maximum attainable MAS rate. However, ^15^N—^1^H distance measurements filter out the ^1^H signals which are not connected to the ^15^N nuclei, allowing direct measure of ^15^N-^1^H dipolar interactions without any overlap of ^1^H resonances.

This article describes a method where high-field ssNMR is used to determine accurate ^15^N—^1^H distances in a series of prototypical salt/cocrystal/continuum systems of the type {PyN⋯H—O—}/{PyN^+^—H⋯O^−^} at natural abundance without any isotopic dilution/enrichment. Of special significance is that we have used natural-abundance samples throughout. Furthermore, the distances obtained from ssNMR data were compared with the distances obtained from single-crystal X-ray diffraction (SCXRD) data.

## 2. Experimental section   

### 2.1. General procedures   

All reagents were purchased from commercial sources and were used without further purification. Fourier Transform infrared (FT–IR) spectra were recorded in ATR mode with a PerkinElmer (Frontier) spectrophotometer (4000–400 cm^−1^). Powder X-ray diffraction (PXRD) data was recorded using a Philips X’pert Pro X-ray powder diffractometer equipped with an X’cellerator detector at room temperature with a scan range 2*θ* = 5–40° and a step size of 0.026°. *X’PertHighScore Plus* values were used to compare the experimental PXRD pattern with the calculated lines from the crystal structure. Differential scanning calorimetry (DSC) was performed on a Mettler Toledo DSC 823e module with the heating rate of 5 K min^−1^ under a nitro­gen atmosphere.

### 2.2. Crystallization method   

Crystallization experiments were carried out under ambient conditions for the four model compounds **SA1**, **SA2**, **CO1** and **CNT1**.

**SA1**: *N*,*N*-Dimethypyridin-4-amine and 3-nitro­benzoic acid were taken in a 1:1 molar ratio in a conical flask and dissolved in a minimum amount of MeOH. Good-quality crystals, suitable for diffraction, were obtained after 4–5 d.

**SA2**: 4-Ethyl­pyridine and 3,5-di­nitro­benzoic acid were taken in a 1:1 molar ratio in a conical flask and dissolved in a minimum amount of MeOH. Good-quality crystals, suitable for diffraction, were obtained after 5–6 d.

**CO1**: 3-Ethyl­pyridine and 4-nitro­benzoic acid were taken in a 1:1 molar ratio in a conical flask and dissolved in a minimum amount of MeOH. Good-quality crystals, suitable for diffraction, were obtained after 6–7 d.

**CNT1**: 4-Methyl­pyridine and 2,3,4,5,6-penta­chloro­phenol were taken in a 1:1 molar ratio in a conical flask and dissolved in a minimum amount of MeOH. Good-quality crystals, suitable for diffraction, were obtained after 6–7d.

### 2.3. Single-crystal X-ray diffraction   

Single-crystal X-ray diffraction (SCXRD) data were collected on a Rigaku Mercury 375/M CCD (XtaLAB mini) using graphite-monochromated Mo *K*α radiation at 298 and 110 K. The data were processed using *CrystalClear* software (Rigaku, 2009 ▸). Some data sets were collected on a Bruker SMART APEX (D8 QUEST) CMOS diffractometer equipped with an Oxford cryosystems N_2_ open-flow cryostat using Mo *K*α radiation. Data integration and data reduction were carried out with the *SAINT-Plus* program (Bruker, 2006 ▸). Structure solution and refinement were executed using *SHELXL97* (Sheldrick, 2008 ▸) embedded in the *WinGX* suite (Farrugia, 1999 ▸) and *OLEX2* (Dolomanov *et al.*, 2009 ▸). Refinement of the coordinates and anisotropic displacement parameters of non-H atoms were performed using the full-matrix least-squares method. H-atom positions were located from difference Fourier maps or calculated using the riding model. However, the H atoms of the protonated pyridine and –COOH groups were located from difference Fourier maps. *PLATON* (Spek, 2009 ▸) was used to prepare material for publication.

### 2.4. ssNMR experimental details   

**Figure 2:**
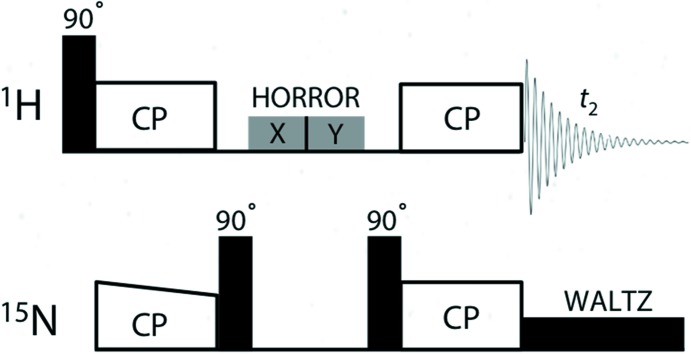
The pulse sequence used to record the two-dimensional inversely proton-detected cross polarization with variable contact-time (invCP-VC) spectra.

The N—H distances/dipolar couplings are measured by two-dimensional inversely proton-detected cross polarization with variable contact-time (invCP-VC) experiments at ultrafast MAS frequencies (60–70 kHz) (Park *et al.*, 2013 ▸; Nishiyama *et al.*, 2016 ▸). In CP-VC experiments, the oscillatory behaviour during CP build-up is observed by monitoring the NMR signal intensities with various contact times of CP (Paluch *et al.*, 2013 ▸, 2015 ▸). The Fourier transformation of the NMR signal intensity with respect to the contact time gives two well-separated narrow peaks/singularities of the Pake-like dipolar powder pattern. Although the overall dipolar powder pattern is very sensitive to experimental imperfections, the separation between two singularities gives a reliable measure of the size of the N—H dipolar interactions (Paluch *et al.*, 2015 ▸). While MAS averages out all the homonuclear (^1^H-^1^H) and heteronuclear (^15^N-^1^H) dipolar interactions, the simultaneous *rf* irradiation during CP on ^1^H and ^15^N hinders the averaging, in effect recoupling the ^15^N-^1^H dipolar interactions (Hartmann & Hahn, 1962 ▸). This results in oscillatory magnetization transfer between ^1^H and ^15^N during CP (Müller *et al.*, 1974 ▸). Generally, this oscillatory behaviour is only observed at ultrafast MAS, since it is obscured by residual ^1^H-^1^H dipolar interactions at moderate MAS rates (Paluch *et al.*, 2015 ▸). The sensitivity is maximized by the introduction of the ^1^H indirect detection approach (Müller, 1979 ▸; Bodenhausen & Ruben, 1980 ▸) into the CP-VC scheme. The initial magnetization is first transferred from ^1^H to ^15^N and then back-transferred to ^1^H magnetization for detection. Since the gyromagnetic ratio of ^1^H is ∼10 times larger than that of ^15^N, the initial magnetization of ^1^H is much greater than that of ^15^N and, therefore, significant sensitivity enhancement can be achieved. This can be implemented in the invCP-VC scheme as shown in Fig. 2 ▸. First, ramped-amplitude cross polarization (RAMP-CP) is used for magnetization transfer from ^1^H to ^15^N (Metz *et al.*, 1994 ▸). This prepares the initial ^15^N magnetization which is indirectly observed at the end of the sequence. It is important to remove the unwanted residual ^1^H magnetization by the homonuclear rotary resonance recoupling (HORROR) sequence on the ^1^H channel (Nielsen *et al.*, 1994 ▸; Ishii *et al.*, 2001 ▸), since more than 99% of ^1^H does not have a ^15^N neighbour in a natural-abundance sample. During HORROR irradiation, the ^15^N magnetization is stored along the *z* axis by a pair of ^15^N 90° pulses, such that time-evolution and transversal relaxation of ^15^N are avoided. ^15^N magnetization is then back-transferred to ^1^H by the second constant CP with variable contact times. Finally, the ^1^H signal is acquired under a weak ^15^N-^1^H heteronuclear WALTZ decoupling irradiation (Shaka *et al.*, 1983 ▸) on ^15^N (Wickramasinghe *et al.*, 2015 ▸). The ^1^H signal intensity thus obtained is modulated by the ^15^N-^1^H dipolar interaction, which is recoupled by the second constant CP, giving dipolar oscillation. The time-domain data thus obtained are Fourier transformed into frequency-domain data in both dimensions. DC correction (subtracting the average of the final one-eighth points from the total data points) in the ^1^H-^15^N dipolar dimension should be applied prior to the Fourier transformation to remove the intense central peak. The peak position in the direct dimension represents the ^1^H chemical shift, whereas the separation of the peaks (Δ) in the indirect dimension reflects the ^15^N-^1^H dipolar coupling, which is converted to an ^15^N—^1^H distance (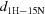) using the following relationship:
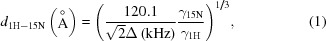
where 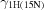 is the gyromagnetic ratio of ^1^H(^15^N) and 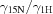 = 0.10136. The equation with the scaling factor of 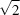 in the invCP-VC experiment is derived from Eq. 2.15 in Schmidt-Rohr & Spiess (1994 ▸). Because of the inverse cubic relationship between the internuclear distance and the magnitude of the dipolar coupling, even for systems with small variations in a distance, the separation of dipolar splitting is significantly different. Consequently, precise N—H internuclear distances can be measured from the experiment. For example, the separations (Δ) for 1.0 and 1.1 Å N—H distances are 8.6 and 6.5 kHz, respectively. Moreover, for longer N—H distances, Δ becomes smaller (Δ = 0.3 kHz for 3 Å); therefore, only those protons with very short ^15^N—^1^H distances show splitting in the indirect dimension and the other protons do not show any splitting.

All the data were collected on a 700 MHz (JNM-ECA700II, Jeol RESONANCE Inc.) NMR spectrometer. For each experiment, about 0.8 mg samples were packed separately into a zirconia sample rotor with an outer diameter of 1 mm. All the experimental parameters used to record the invCP-VC experiments on **CO1**, **CNT1**, **SA2** and **SA1** are given in Table 1 ▸. DQ Hartmann–Hahn CP matching conditions (*ν*_1H_ + *ν*_15N_ = *ν*_R_) were used for all four samples (Laage *et al.*, 2009 ▸). Optimization of the DQ CP condition was carried out using ^15^N_3_-labelled l-histidine·HCl·H_2_O or ^13^C_3_,^15^N-labelled l-alanine by maximizing the ^1^H NMR spectra observed with the sequence shown in Fig. 2 ▸ with a fixed second contact time (typically 1 ms). The experimental conditions can be further verified by observing the invCP-VC spectra of a ^15^N_3_-labelled l-histidine sample. Two N—H protons of the imidazole ring should give splittings of 6.9 and 7.6 kHz if all the experimental conditions are properly adjusted.

## 3. Results and discussion   

**Figure 3:**
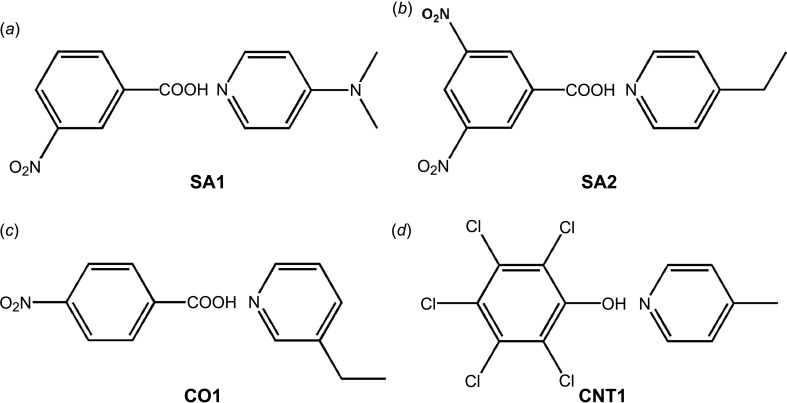
Schematic representation of the compounds used in the present study, showing (*a*) **SA1** (3-nitro­benzoic acid and *N*,*N*-dimethypyridin-4-amine), (*b*) **SA2** (3,5-di­nitro­benzoic acid and 4-ethyl­pyridine), (*c*) **CO1** (4-nitro­benzoic acid and 3-ethyl­pyridine) and (*d*) **CNT1** (penta­chloro­phenol and 4-methyl­pyridine).

For this study, we have prepared four solid forms composed of 3-nitro­benzoic acid and *N*,*N*-dimethypyridin-4-amine (**SA1**) (Saha *et al.*, 2015 ▸), 3,5-di­nitro­benzoic acid and 4-ethyl­pyridine (**SA2**), and 4-nitro­benzoic acid and 3-ethyl­pyridine (**CO1**). We also examined the well-studied case of penta­chloro­phenol and 4-methyl­pyridine (**CNT1**) (Malarski *et al.*, 1987 ▸; Steiner *et al.*, 2001 ▸) (Fig. 3 ▸). All the solid forms were crystallized and characterized by DSC, FT–IR spectroscopy, PXRD, SCXRD and ssNMR. In order to confirm the solid form, *i.e.* the salt/cocrystal/continuum, SCXRD data were collected at room temperature and at 110 K.

The invCP-VC experiment has several advantages com­pared to previously reported heteronuclear distance measurement methods (Ramamoorthy *et al.*, 1999 ▸; Ladizhansky & Vega, 2000 ▸; van Rossum *et al.*, 2000 ▸). These include: (i) accurate estimation of N—H distances [due to the larger dipolar scaling factor (*K*_sc_ = 1/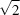)]; (ii) straightforward experimental settings; (iii) robustness towards experimental imperfections, such as *rf* inhomogeneity, Hartmann–Hahn mismatch, *rf* offset and chemical shift anisotropies; (iv) higher sensitivity due to ^1^H detection; and (v) the small sample volume (typically less than 1 mg). While the ^15^N—^1^H distance measurement in a natural-abundance amino acid using the invCP-VC method was demonstrated earlier, the level of difficulty in the measurement is higher for multi­component systems, especially for cocrystals that are associated with longer ^15^N—^1^H distances. In multicomponent systems, a larger number of ^1^H resonances are expected than in a small amino acid. This results in the lower sensitivity and potential signal overlaps. The low sensitivity is partially overcome by the high magnetic field. An additional sensitivity improvement was achieved simply by applying a large number of transients. It typically took four to five days to collect each set of data (Table 1 ▸). The limited spectral resolution of ^1^H nuclei even at an ultrafast MAS rate potentially produces ^1^H resonance overlaps. Fortunately, invCP-VC experiments on the ^15^N—^1^H system filtered out ^1^H signals from atoms which are not bonded directly to ^15^N. Thus, the signal overlaps can easily be avoided in salt/cocrystal/continuum systems which typically include only one N—H hydrogen-bonding pair. Longer ^15^N—^1^H distances result in smaller ^15^N-^1^H dipolar interactions. This may introduce the effect of remote ^15^N-^1^H dipolar interactions which are usually suppressed by the strongest ^15^N-^1^H dipolar interactions. This effect depends on numerous factors, including the size of each dipolar interaction and the relative orientation of each N—H vector. We calculated the invCP-VC spectrum of **CO1**, which shows the longest N—H distances, to evaluate the effects of the second and third nearest-neighbour H atoms. It was shown that these remote N-H dipolar interactions only broaden the invCP-VC spectra and do not affect the peak positions, *i.e.* ^15^N—^1^H distances (Fig. S14 in the supporting information). Therefore, we conclude that one may safely rely on dipolar splitting to obtain ^1^H—^15^N distances, even for cocrystals.

One of the major requirements of the two-dimensional invCP-VC ^1^H→^15^N→^1^H experiment with natural-abundance samples is to have as short a ^1^H spin lattice relaxation time (*T*_1_) as possible. However, in pharmaceutical cocrystals, *T*_1_ is generally long for N—H protons. The long *T*_1_ relaxation time results in impractical total experimental times and limits the application of two-dimensional invCP-VC ^1^H→^15^N→^1^H experiments on actual samples. The application of a rotor-synchronized train of 180° pulses (*i.e.* RFDR or radio-frequency driven recoupling) during the recycle period (Ye *et al.*, 2014 ▸) and/or the addition of a paramagnetic dopant (Wickramasinghe *et al.*, 2007 ▸) to the system can be used to reduce *T*_1_ values somewhat. For our purpose, we have used the RFDR-based approach in three of the samples (**CO1**, **SA1** and **SA2**) to reduce the *T*_1_ relaxation times. Besides, paramagnetic doping was avoided to preserve the sample purity. While the *T*_1_ relaxation time for an NH proton in the case of **CNT1** is also very long (70 s), the uniform ^1^H *T*_1_ relaxation hampers the application of the RFDR pulse train and data were collected without RFDR irradiation. The one-dimensional ^1^H→^15^N→^1^H-filtered spectra of all four samples under study give isolated N—H resonances (Fig. S15 in the supporting information). All other unwanted peaks are completely suppressed. In other words, this experiment provides a method for the precise assignment of N—H proton resonances in cases where the signals are overlapped with other ^1^H resonances (C—H protons) and allows a more accurate and reliable measurement of N—H distances. This highlights the additional advantages of invCP-VC experiments for multicomponent systems where severe overlap of ^1^H resonances is expected.

**Figure 4:**
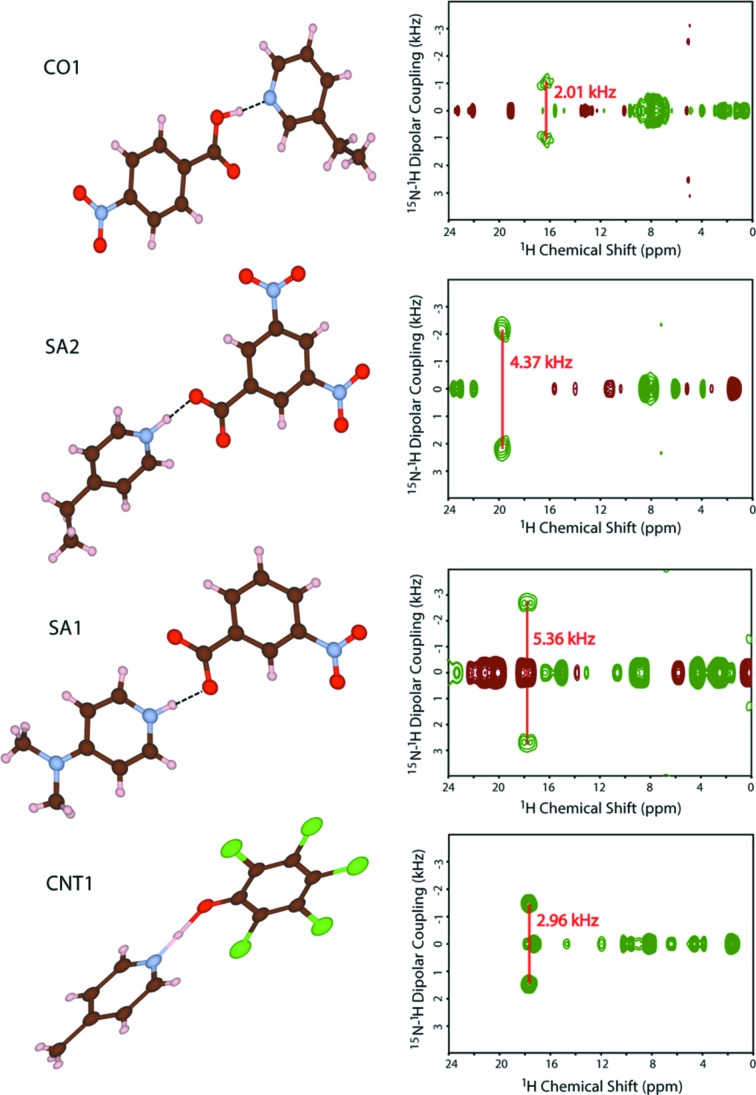
The molecular structures and the two-dimensional invCP-VC spectra (^15^N-^1^H dipolar couplings *versus* ^1^H chemical shift) of **SA1**, **SA2**, **CO1** and **CNT1**.

The two-dimensional invCP-VC spectra were plotted with the horizontal and vertical axes representing the ^1^H chemical shift and the size of the ^15^N-^1^H dipolar couplings, respectively (Fig. 4 ▸). The above-mentioned procedure overcomes the difficulties, including low abundance and thus sensitivity of ^15^N, small ^15^N-^1^H dipolar coupling, complex multicomponent systems and long ^1^H *T*_1_ relaxation time, and clearly gives splitting in the indirect dimension. The observed separations between two singularities/^15^N-^1^H dipolar splittings for **SA1**, **SA2**, **CO1** and **CNT1** were found to be 5.36, 4.37, 2.01 and 2.96 kHz, respectively, corresponding to ^15^N—^1^H distances of 1.17, 1.25, 1.62 and 1.43 Å. From SCXRD at 298 K, the ^15^N—^1^H distances were 1.01, 1.20, 1.54 and 0.99 Å (without normalization), and at 110 K, they were 0.99, 1.18, 1.57 and 1.17 Å for **SA1**, **SA2**, **CO1** and **CNT1**, respectively (Table 2 ▸). Crystal data, data collection and structure refinement details are summarized in Table 3 ▸.

X-ray crystallography is a powerful and widely accepted technique for structure determination. Since X-rays are scattered by the electrons of an atom, the results of an X-ray-based structure determination give the centroids of the electron density, which correspond to the centres of the nuclei in heavier atoms. In securing light-atom positions, and especially H-atom positions, X-ray diffraction has its limitations. The electron density of an H atom is not centred around the H-atom nucleus, but is aspherically displaced towards the covalently bonded heavier atom (X—H⋯*A*—*Y*). As a result, X-ray-determined *X*−H distances generally appear to be shorter than the true internuclear distance (Desiraju & Steiner, 1999 ▸; Lusi & Barbour, 2011 ▸). This problem can be avoided by the use of neutron diffraction (ND) analysis, in which the positional and anisotropic displacement parameters of the H atoms can be refined. But the distance (*X*−H) derived from ND analysis corresponds to the internuclear distance, since the scattering centres are the atomic nuclei.

In this regard, a comparison between the ND- and ssNMR-derived *X*−H distance would appear to be significant. In fact, there are several important reports where the NMR-derived distances of l-histidine·HCl·H_2_O were compared with the ND-derived distances (Zhao *et al.*, 2001 ▸; Paluch *et al.*, 2015 ▸). We have also demonstrated that the invCP-VC method applied to l-histidine·HCl·H_2_O gives as reliable an internuclear N—H distance as the ND method (Nishiyama *et al.*, 2016 ▸). However, ND is expensive and limited by availability. It also requires a large amount of samples or a large-sized crystal, which is often difficult to grow. Therefore, alternatively, a comparison of the neutron-normalized (NN) X-ray distance, in lieu of the neutron-derived distance, with the ssNMR-derived distance can be considered reliably meaningful. It should be noted that the NN X-ray distance is an outcome of the analysis of available XRD and ND crystal structure data in the Cambridge Structural Database (Groom *et al.*, 2016 ▸), where the X-ray-derived distance is extended by shifting the *X*−H bond vector to the average neutron-derived distance (Allen & Bruno, 2010 ▸). This is a widely used procedure.

The ssNMR *X*−H distances of **CO1** (1.62 Å) and **SA1** (1.17 Å) show a fair agreement with the NN X-ray distances of **CO1** (1.65 Å) and **SA1** (1.01 Å). The observed differences in the measured ^15^N—^1^H distances from the two methods (XRD/ND and ssNMR) could be attributed to: (i) inaccurate proton positions due to a low scattering cross sectional area of the H atoms, (ii) different timescales of measurements (tens of µs in NMR and ps in XRD/ND) and, therefore, averaging of the libratory motions at different timescales, and (iii) dissimilar distance dependence (1/*r* ^3^ in NMR and 1/*r* in XRD/ND) which ultimately resulted in a longer distance for NMR compared to XRD (Ishii *et al.*, 1997 ▸). Since the current approach gives the internuclear distances directly, unlike diffraction-based methods, where the distances are calculated from the positions of the nuclei, ssNMR-based approaches are appropriately com­plementary to the other methods.

From ssNMR, the ^15^N—^1^H distance in **SA1** is 1.17 Å and is the smallest in comparison to the corresponding distances in **SA2**, **CNT1** and **CO1**. This clearly suggests that **SA1** is a ‘salt’ in which complete transfer of the proton across the hydrogen bond from acid to pyridine has taken place. Similarly, the longer distance observed for **CO1** (1.62 Å) clearly rules out any possibility of the transfer of the proton from the acid to pyridine, confirming that **CO1** is a ‘cocrystal’. Interestingly, **SA2**, which is an adduct of a strong acid and a strong base, is a case of a continuum, despite having Δp*K*_a_ > 3, with an ^15^N—^1^H distance of 1.25 Å from ssNMR, half of the N—O distance (2.54 Å by SCXRD at both temperatures). **CNT1** shows the importance of locating accurate proton positions in these classifications. In this example, unlike in the previously discussed continuum scenario, the distance obtained from SCXRD and ssNMR were not comparable. The SCXRD distance was completely misleading due to poor data quality. From the difference Fourier map, the residual electron density is delocalized over a region between the two heavy atoms (O and N) and the proton position had to be fixed at 0.99 Å (SCXRD at 298 K, Rigaku XtaLAB mini). Further refinements were not sustained. Alternatively, the SCXRD measurement was carried out on a Bruker SMART APEX (D8 QUEST) CMOS diffractometer. The N—H distance was 1.65 Å at room temperature, *i.e.* 0.18 Å longer than obtained by Steiner *et al.* (2001 ▸). In other words, locating the proton position in a salt–cocrystal continuum is ambiguous from SCXRD data as it is somewhat machine dependent. Neutron normalization of the X-ray distance, in this regard, is also not appropriate since neutron normalization adjusts the H-atom position to an average distance, and practically ignores the polarization effect caused by the acceptor atom in a strongly hydrogen-bonded system. In this regard, a comparative study with ND data of a system where large crystals could be obtained would appear to be the next step. None of the compounds studied here was available in the form of large crystals.

The ^15^N—^1^H distance of **CNT1** obtained by invCP-VC ssNMR is 1.43 Å at room temperature, which suggests that **CNT1** behaves more like a ‘cocrystal’ at room temperature (N⋯O = 2.54 Å by SCXRD). Previous studies on **CNT1** with variable-temperature time-of-flight neutron diffraction shows that the N—H distance increases with temperature and that it is 1.306 Å at 200 K (Steiner *et al.*, 2001 ▸). Therefore, at room temperature, the N—H distance is expected to be longer than 1.306 Å. As a result, the N—H distance at room temperature determined by ssNMR can be considered to be as reliable as neutron diffraction data. In this situation, the two-dimensional invCP-VC method turns out to be a significant tool for location of precise proton positions through dipolar coupling, especially in proton-disordered systems. In addition, the present ssNMR technique is advantageous as it can be performed on microcrystalline, or even amorphous, samples with laboratory-based NMR equipment.

## 4. Conclusions   

We have carried out two-dimensional inversely proton-detected CP-VC ssNMR measurements at fast MAS to determine N—H distances with naturally abundant ^15^N nuclei in multicomponent solid forms. N—H distances vary with the length of the hydrogen bond between the two individual components of a salt, cocrystal or continuum, and these distances were measured through two well-separated singularities of the Pake-like dipolar powder pattern. The measured distances can be easily used to locate the proton positions in such systems and hence a clear distinction between salt, cocrystal and continuum may be established. The technique will be useful where the Δp*K*_a_ rule has limitations, especially in the range 1 < Δp*K*_a_ < 3. We believe that the method presented in this work will have an impact on the pharmaceutical industry. Further, the method can be utilized for microcrystalline samples where obtaining a single crystal is difficult. Our future studies will be directed towards the implementation of this technique in more complex systems, such as to differentiate polyamorphous solid forms.

## Supplementary Material

Crystal structure: contains datablock(s) SA-1-LT, SA-1-RT, SA-2-LT, SA-2-RT, CO-1-LT, CO-1-RT, CNT-1-LT, CNT-1-RT, CNT1-LT, CNT1-RT. DOI: 10.1107/S205225251700687X/lq5005sup1.cif

Spectra and hydrogen-bonding details. DOI: 10.1107/S205225251700687X/lq5005sup2.pdf

Structure factors: contains datablock(s) data. DOI: 10.1107/S205225251700687X/lq5005CNT-1-LTsup3.hkl

Structure factors: contains datablock(s) data. DOI: 10.1107/S205225251700687X/lq5005CNT-1-RTsup4.hkl

Structure factors: contains datablock(s) cnt1-lt1. DOI: 10.1107/S205225251700687X/lq5005CNT1-LTsup5.hkl

Structure factors: contains datablock(s) cnt1-rt1. DOI: 10.1107/S205225251700687X/lq5005CNT1-RTsup6.hkl

Structure factors: contains datablock(s) datap1. DOI: 10.1107/S205225251700687X/lq5005CO-1-LTsup7.hkl

Structure factors: contains datablock(s) datatri. DOI: 10.1107/S205225251700687X/lq5005CO-1-RTsup8.hkl

Structure factors: contains datablock(s) datac2c. DOI: 10.1107/S205225251700687X/lq5005SA-1-LTsup9.hkl

Structure factors: contains datablock(s) datam. DOI: 10.1107/S205225251700687X/lq5005SA-1-RTsup10.hkl

Structure factors: contains datablock(s) sa5-meoh-lt1. DOI: 10.1107/S205225251700687X/lq5005SA-2-LTsup11.hkl

Structure factors: contains datablock(s) sa5-meoh1. DOI: 10.1107/S205225251700687X/lq5005SA-2-RTsup12.hkl

CML file for SA1. DOI: 10.1107/S205225251700687X/lq5005SA-1sup13.cml

CML file for SA2. DOI: 10.1107/S205225251700687X/lq5005SA-2sup14.cml

CML file for CO1. DOI: 10.1107/S205225251700687X/lq5005CO-1sup15.cml

CML file for CNT1. DOI: 10.1107/S205225251700687X/lq5005CNT1sup16.cml

CML file for CNT-1. DOI: 10.1107/S205225251700687X/lq5005CNT-1sup17.cml

CCDC references: 1529546, 1529544, 1529545, 1529547, 1529549, 1529550, 1529542, 1529548, 1529541, 1529543

## Figures and Tables

**Table 1:** All the experimental parameters used to record the invCP-VC experiments

Compound	Spectrometer frequency (MHz)	MAS rate (kHz)	^1^H 90° (µs)	^15^N 90° (µs)	Scans	Recycle delay (s)	Contact time (First CP)	Contact time (Second CP)	Total experiment time (days)
**SA1**	700	60	0.9	2.2	288	70	1.0 ms	10 µs–0.71 ms (15 increments)	3.5
**SA2**	700	70	0.9	2.2	736	20	1.5 ms	30 µs–1.02 ms (34 increments)	5.8
**CO1**	700	70	1	5	320	8	2.0 ms	0 µs–1.52 ms (151 increments)	4.5
**CNT1**	700	70	0.7	5	136	70	2.0 ms	0 µs–1 ms (51 increments)	5.0

**Table 2:** ^15^N—^1^H distance (Å) measurements by SCXRD^*a*^ and ssNMR for the four investigated solid forms

Compound	SCXRD (298 K)	SCXRD (298 K) Normalized	SCXRD (110 K)	SCXRD (110 K) Normalized	ssNMR
**SA1**	1.01 (3)	1.01	0.99 (2)	1.01	1.17
**SA2**	1.20 (3)	1.01	1.18 (3)	1.01	1.25
**CO1**	1.54 (4)	1.65	1.57 (3)	1.64	1.62
**CNT1**	0.99 (9)	1.01	1.17 (6)	1.01	1.43

Note: (*a*) data sets were collected on a Rigaku XtaLAB mini diffractometer.

**Table 3:** Crystallographic parameters of compounds **SA1**, **SA2**, **CO1** and **CNT1**

	**SA1** (RT)	**SA1** (LT)	**SA2** (RT)	**SA2** (LT)	**CO1** (RT)
Chemical formula	C_14_H_15_N_3_O_4_	C_14_H_15_N_3_O_4_	C_14_H_13_N_3_O_6_	C_14_H_13_N_3_O_6_	C_14_H_14_N_2_O_4_
*M*r	289.29	289.29	319.27	319.27	274.27
Crystal system	Monoclinic	Monoclinic	Monoclinic	Monoclinic	Triclinic
Space group	*C*2/*c*	*C*2/*c*	*P*2_1_/*c*	*P*2_1_/*c*	*P* 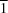
Temperature (K)	298	110	298	110	298
*a* (Å)	28.86 (2)	28.685 (2)	8.574 (9)	8.439 (6)	6.730 (2)
*b* (Å)	6.791 (5)	6.783 (3)	14.346 (2)	14.091 (9)	7.186 (2)
*c* (Å)	14.243 (1)	13.975 (7)	12.190 (1)	12.167 (8)	14.298 (3)
α (°)	90	90	90	90	88.158 (6)
β (°)	95.410 (1)	94.175 (7)	94.440 (1)	95.500 (1)	88.340 (6)
γ (°)	90	90	90	90	78.636 (5)
*V* (Å^3^)	2779 (3)	2712 (2)	1495 (3)	1440.0 (2)	677.4 (3)
*Z*	8	8	4	4	2
*D*_calcd_(Mg m^−3^)	1.383	1.417	1.419	1.472	1.345
μ (mm^−1^)	0.103	0.106	0.113	0.117	0.100
*F*(000)	1216	1216	664	664	288
Total reflections	12517	12292	13680	13152	5680
Unique reflections	2729	2662	2936	2815	2634
Observed reflections [*I* > 2σ(*I*)]	2388	2507	2597	2683	1543
*R*_int_	0.059	0.060	0.080	0.097	0.040
*R*_1_ [*I* > 2σ(*I*)]	0.0498	0.0383	0.0555	0.0400	0.0646
*wR*_2_	0.1414	0.1119	0.1737	0.1248	0.1894
Completeness (%)	99.6	99.7	99.9	99.6	99.2
Goodness-of-fit	1.081	1.099	1.135	1.143	1.037
CCDC No.	1529544	1529546	1529547	1529545	1529550
Diffractometer	Rigaku	Rigaku	Rigaku	Rigaku	Rigaku
					
	**CO1** (LT)	**CNT1** (RT)	**CNT1** (LT)	**CNT1** (RT)	**CNT1** (LT)
Chemical formula	C_14_H_14_N_2_O_4_	C_12_H_8_Cl_5_NO	C_12_H_8_Cl_5_NO	C_12_H_8_Cl_5_NO	C_12_H_8_Cl_5_NO
*M*r	274.27	359.44	359.44	359.44	359.44
Crystal system	Triclinic	Triclinic	Triclinic	Triclinic	Triclinic
Space group	*P* 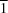	*P* 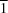	*P* 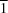	*P* 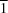	*P* 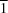
Temperature (K)	110	298	110	298	110
*a* (Å)	6.631 (5)	7.389 (8)	7.316 (6)	7.386 (8)	7.338 (8)
*b* (Å)	7.032 (6)	8.922 (8)	8.942 (8)	8.920 (1)	8.899 (9)
*c* (Å)	14.216 (1)	12.014 (1)	11.763 (9)	12.023 (1)	11.825 (1)
α (°)	87.967 (2)	69.82 (3)	70.15 (4)	69.770 (3)	69.945 (5)
β (°)	88.58 (3)	85.61 (4)	84.67 (4)	85.869 (3)	85.055 (5)
γ (°)	80.207 (2)	76.26 (4)	76.24 (4)	76.324 (4)	76.133 (5)
*V* (Å^3^)	652.6 (9)	722.1 (1)	703.0 (1)	722.1 (1)	704.2 (1)
*Z*	2	2	2	2	2
*D*_calcd_(Mg m^−3^)	1.396	1.653	1.698	1.653	1.695
μ (mm^−1^)	0.104	0.993	1.020	0.993	1.018
*F*(000)	288	360	360	360	360
Total reflections	6144	6793	6521	6870	11629
Unique reflections	2555	2815	2741	2799	2756
Observed reflections [*I* > 2σ(*I*)]	2366	2152	2421	1751	2106
*R*_int_	0.071	0.063	0.054	0.039	0.052
*R*_1_ [*I* > 2σ(*I*)]	0.0427	0.0505	0.0402	0.0466	0.0396
*wR*_2_	0.1338	0.1943	0.1513	0.1288	0.1034
Completeness (%)	99.6	99.6	99.4	98.9	99.7
Goodness-of-fit	1.105	1.155	1.299	1.040	1.102
CCDC No.	1529549	1529548	1529542	1529543	1529541
Diffractometer	Rigaku	Rigaku	Rigaku	Bruker	Bruker
