# Supplementary material for: Exploring the salt–cocrystal continuum with solid-state NMR using natural-abundance samples: implications for crystal engineering
Source: IUCrJ. 2017 Jun 5;4(Pt 4):466–75. doi: 10.1107/S205225251700687X (PMC5571809; doi:10.1107/S205225251700687X)
Supplement: Supplementary file 2 [file m-04-00466-sup2.pdf]

IUCrJ

Volume 4 (2017)

Supporting information for article:

Exploring the salt–cocrystal continuum with solid-state NMR using natural-abundance samples: implications for crystal engineering

Lalit Rajput, Manas Banik, Jayasubba Reddy Yarava, Sumy Joseph, Manoj Kumar Pandey, Yusuke Nishiyama and Gautam R. Desiraju

Exploring the salt-cocrystal continuum with solid-state NMR using natural abundance samples: implications for crystal engineering

Lalit Rajput[a†], Manas Banik[a†], Jayasubba Reddy Yarava[b1], Sumy Joseph[a], Manoj Kumar Pandey[b,c,d], Yusuke Nishiyama[b,c*] and Gautam R. Desiraju[a*]

Contents

S1: PXRD of **SA1**, **SA2**, **CO1**, and **CNT1**

S2: DSC of **SA1**, **SA2**, **CO1**, and **CNT1**

S3: FTIR of **SA1**, **SA2**, **CO1**, and **CNT1**

S4: Hydrogen bonding parameters of **SA1**, **SA2**, **CO1**, and **CNT1**

S5: ORTEP diagram for **SA1**, **SA2**, **CO1**, and **CNT1** (Rigaku)

S6: Calculated invCP-VC spectra of **CO1**

S7: ssNMR 1D spectra of **SA1**, **SA2**, **CO1**, and **CNT1**

S1. PXRD of SA1, SA2, CO1, and CNT1.

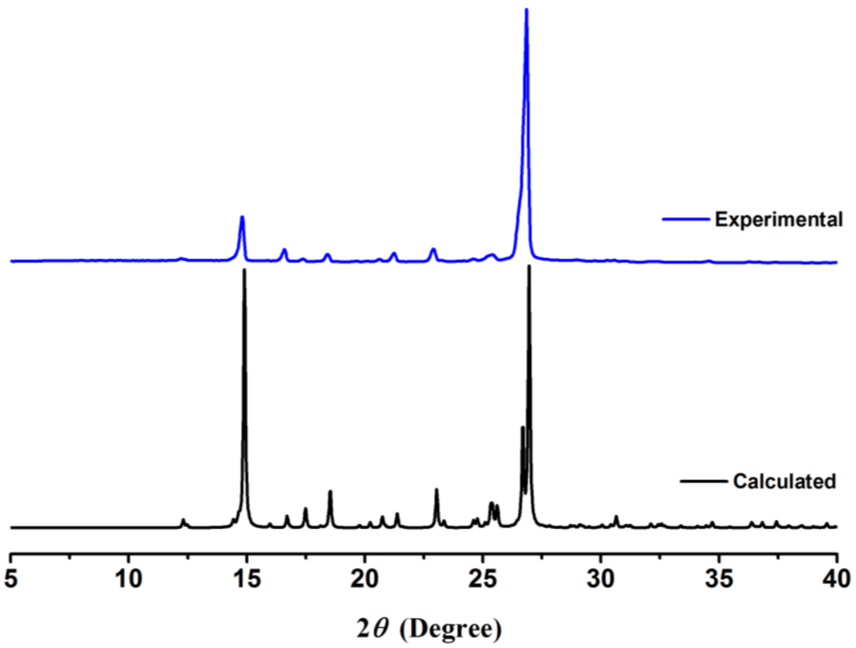

Figure S1 PXRD of **SA1**.

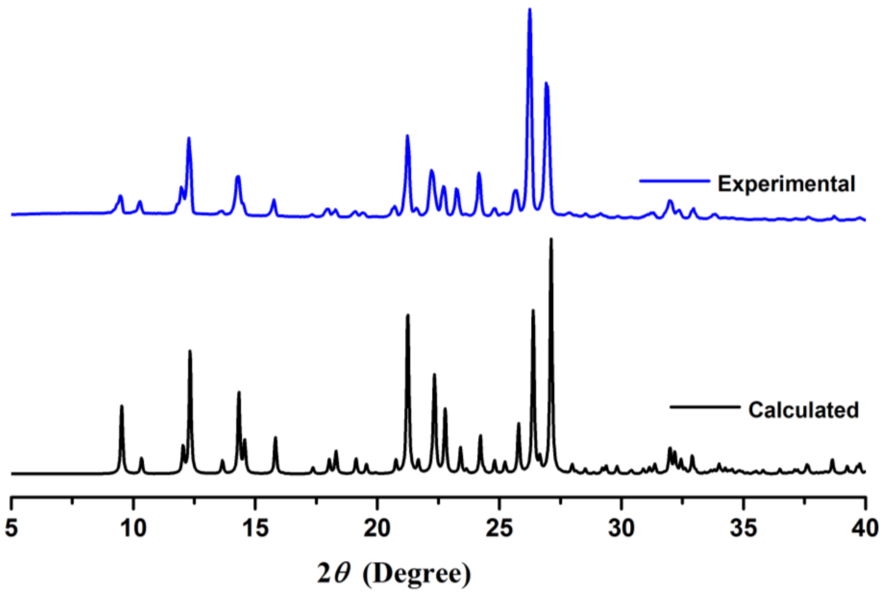

Figure S2 PXRD of **SA2**.

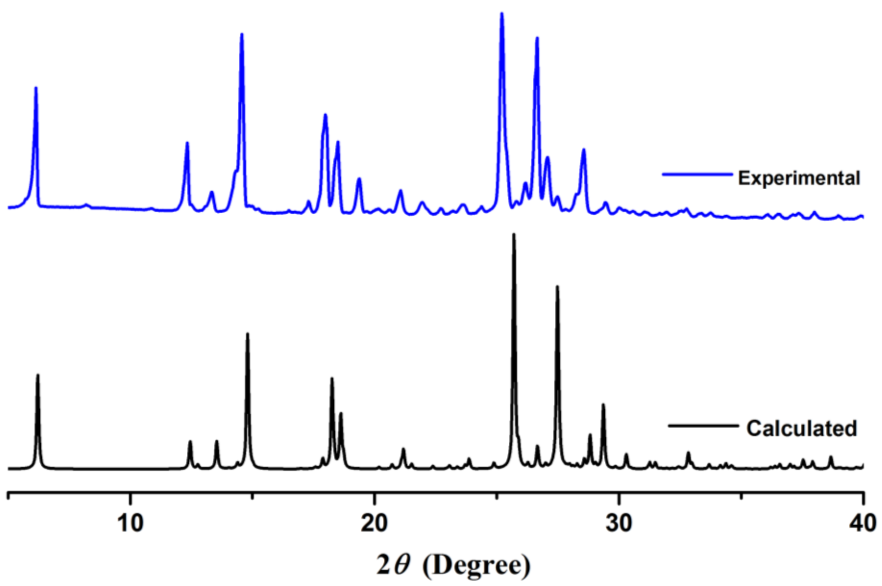

Figure S3 PXRD of **CO1**.

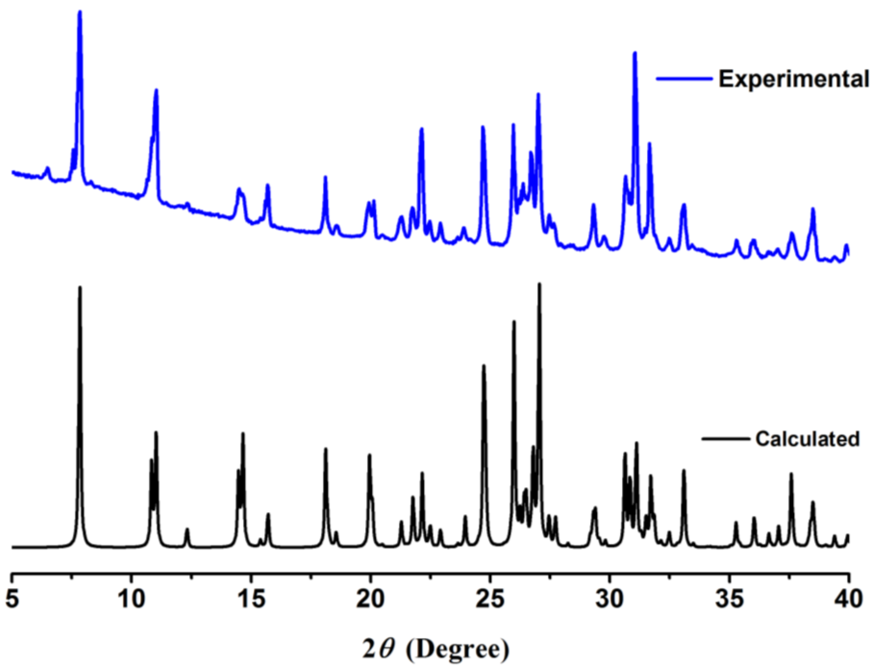

Figure S4 PXRD of **CNT1**.

S2. DSC of SA1, SA2, CO1, and CNT1.

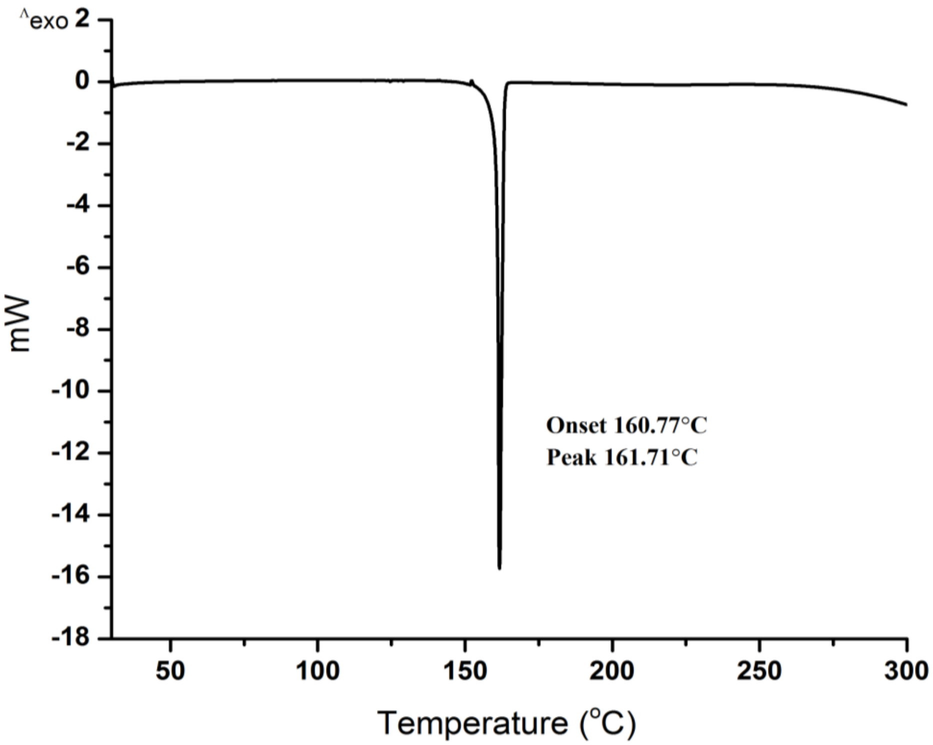

Figure S5 DSC of **SA1**.

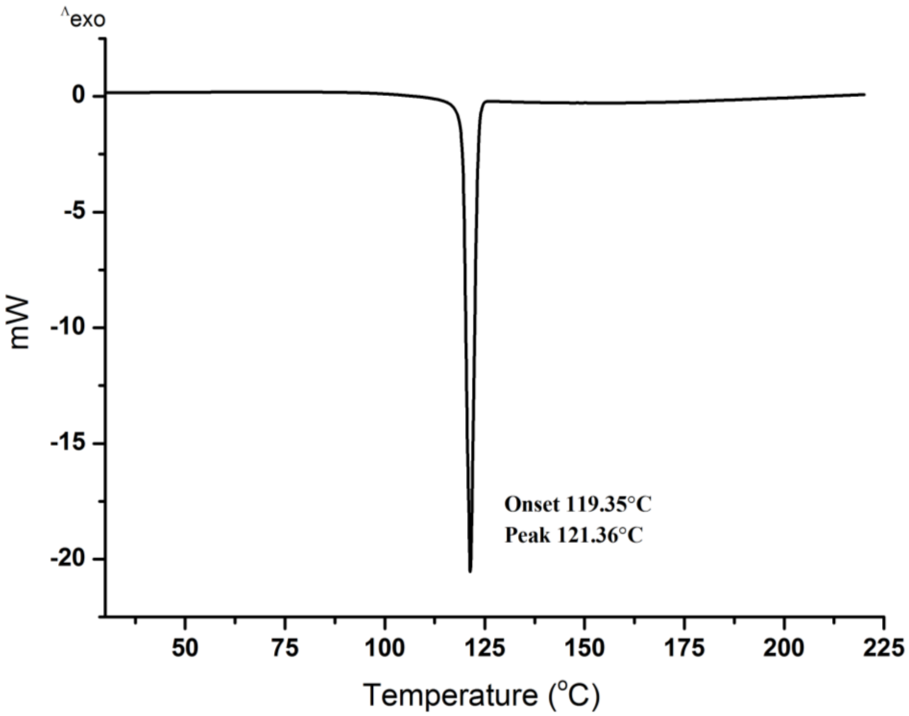

Figure S6 DSC of **SA2**.

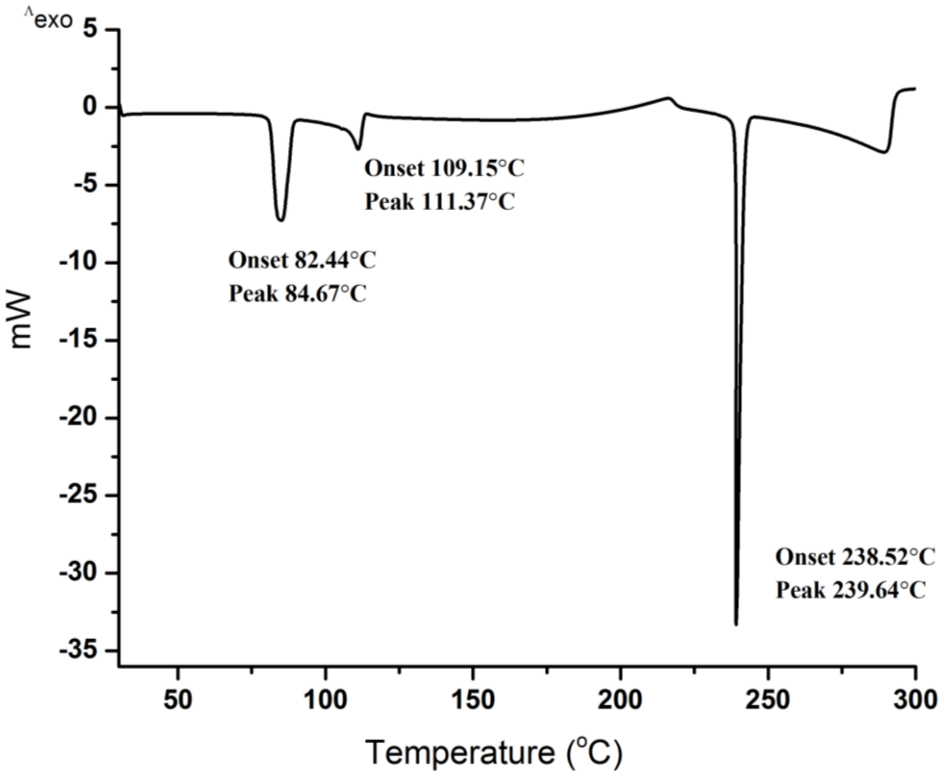

Figure S7 DSC of **CO1**.

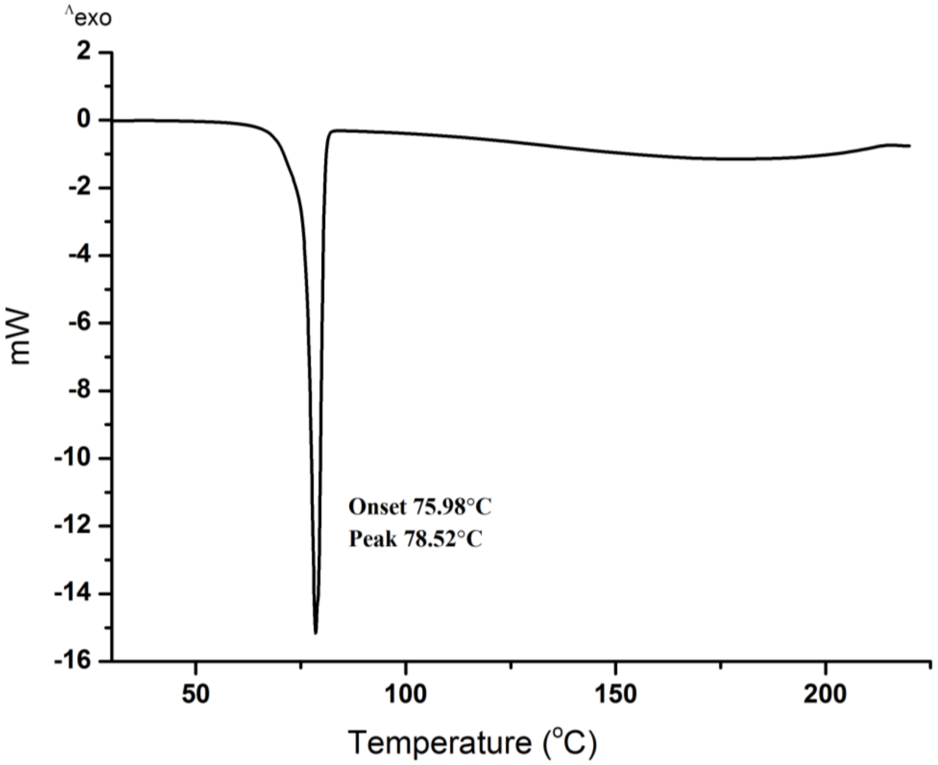

Figure S8 DSC of **CNT1**.

S3. FTIR of SA1, SA2, CO1, and CNT1

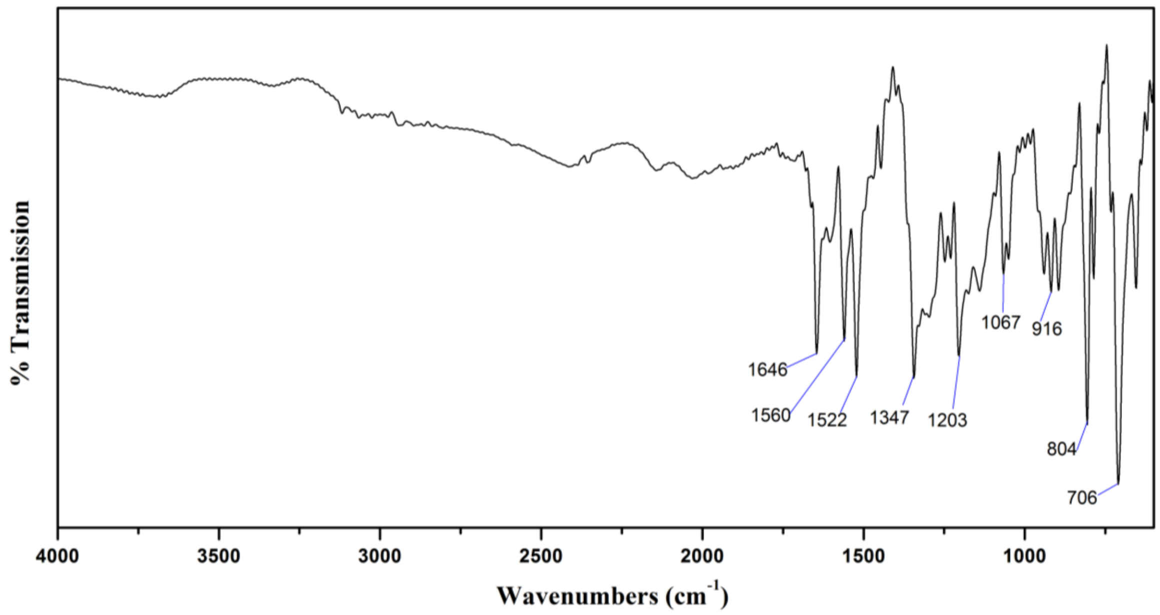

Figure S9 FTIR of **SA1**.

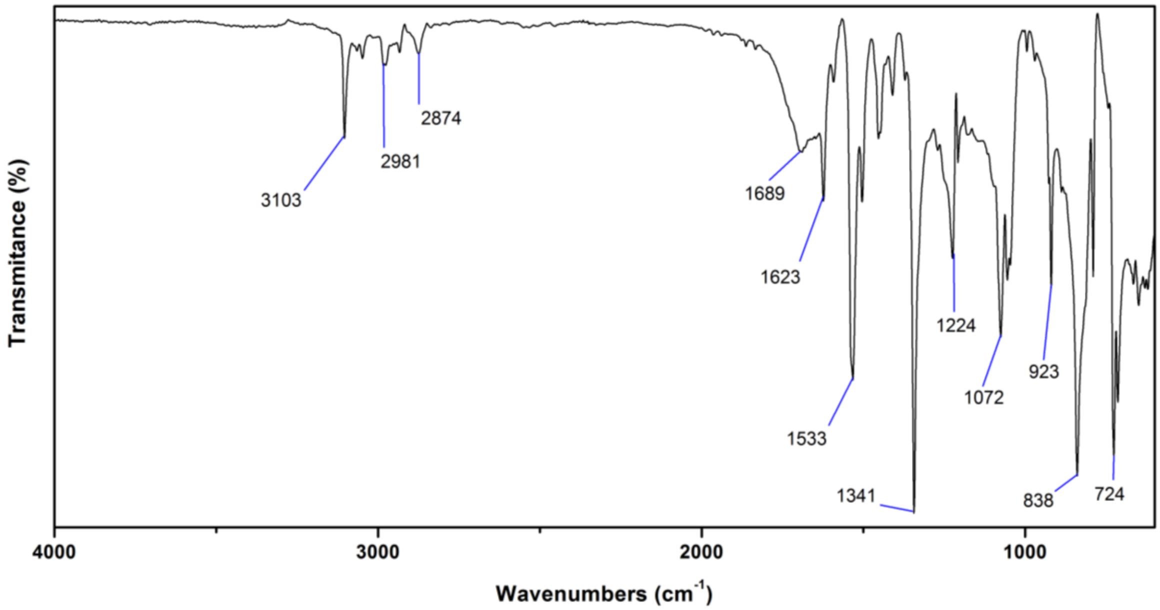

Figure S10 FTIR of **SA2**.

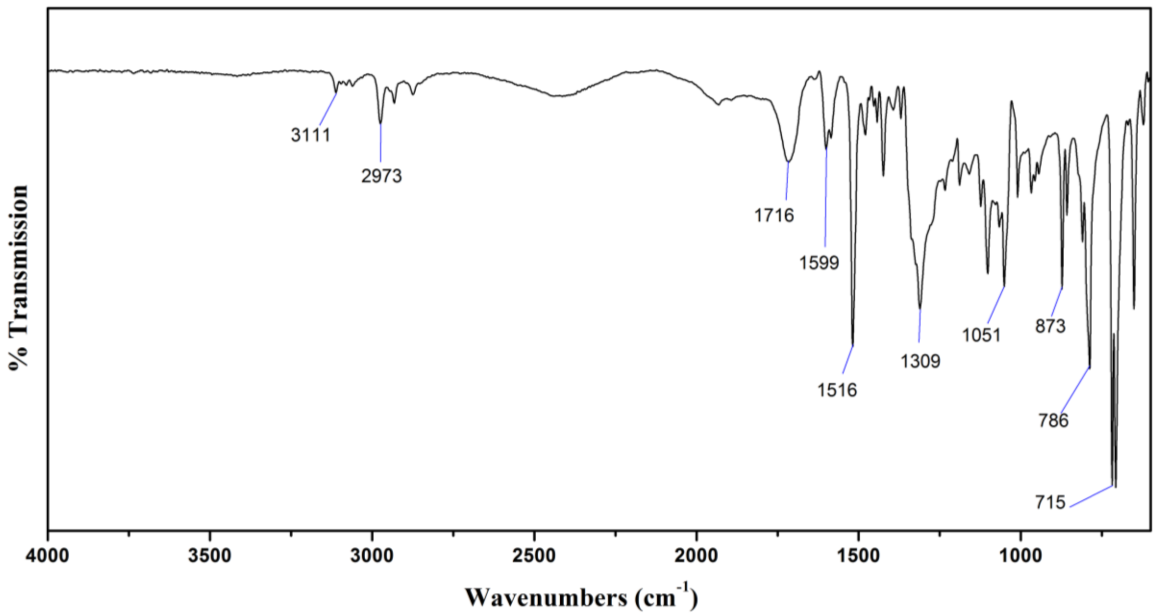

Figure S11 FTIR of **CO1**.

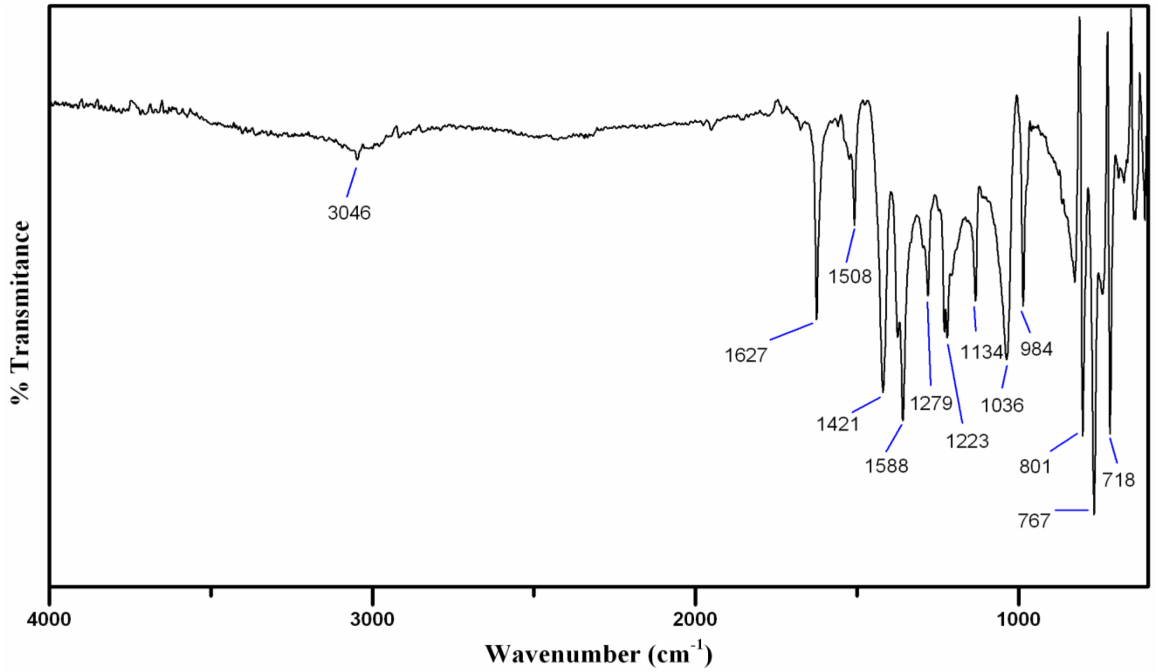

Figure S12 FTIR of **CNT1**.

S4. Hydrogen bonding parameters of SA1, SA2, CO1, and CNT1

Table S1 Hydrogen bond geometrical parameters (Rigaku) of **SA1, SA2, CO1, and CNT1**.

Compound	Interaction	D–H/ Å	H···A/ Å	D···A/ Å	∠D–H···A/ º	Symmetry
**SA1(RT)**	N(1)–H(1N)···O(2)	1.01(3)	1.60(3)	2.611(3)	176(2)	1/2-x,1/2+y,1/2-z
	C(1)–H(1)···O(1)	0.98(2)	2.46(2)	3.214(3)	133.1(2)	1/2-x,1/2+y,1/2-z
**SA1(LT)**	N(1)–H(1N)···O(2)	0.99(2)	1.62(2)	2.613(2)	177.3(2)	1/2-x,-1/2+y,1/2-z
	C(1)–H(1)···O(1)	0.982(1)	2.478(1)	3.212(2)	131.3(1)	1/2-x,-1/2+y,1/2-z
**SA2(RT)**	N(1)–H(1N)···O(1)	1.20(3)	1.35(3)	2.556(3)	178(3)	-1+x,1/2-y,1/2+z
	C(1)–H(1)···O(2)	0.93(2)	2.51(3)	3.197(4)	131(2)	-1+x,1/2-y,1/2+z
**SA2(LT)**	N(1)–H(1N)···O(1)	1.18(3)	1.37(3)	2.545(2)	179(3)	x,1/2-y,1/2+z
	C(1)–H(1)···O(2)	0.940(2)	2.443(2)	3.160(3)	133.1(1)	x,1/2-y,1/2+z
**CO1(RT)**	O(1)–H(1A)···N(1)	1.10(4)	1.54(4)	2.629(3)	171(3)	1+x,y,z
	C(1)–H(1)···O(2)	0.93(3)	2.41(3)	3.172(3)	140(2)	-1+x,y,z
**CO1(LT)**	O(1)–H(1A)···N(1)	1.06(3)	1.57(3)	2.620(3)	173(2)	x,y,1+z
	C(1)–H(1)···O(2)	0.975(1)	2.374(2)	3.144(3)	135.4(1)	x,y,-1+z
**CNT1(RT)**	N(1)–H(1N)···O(1)	0.99(9)	1.66(1)	2.550(5)	148(9)	
**CNT1(LT)**	N(1)–H(1)···O(1)	1.17(6)	1.36(6)	2.525(4)	175(6)	

Table S2 Hydrogen bond geometrical parameters of **SA1, SA2, CO1, and CNT1** (O–H, N–H and C–H distances are neutron normalized).

Compound	Interaction	D–H/ Å	H···A/ Å	D···A/ Å	∠D–H···A/ º	Symmetry
**SA1(RT)**	N(1)–H(1N)···O(2)	1.01	1.60	2.611(3)	176	1/2-x,1/2+y,1/2-z
	C(1)–H(1)···O(1)	1.08	2.39	3.214(3)	131	1/2-x,1/2+y,1/2-z
**SA1(LT)**	N(1)–H(1N)···O(2)	1.01	1.60	2.613(2)	177	1/2-x,-1/2+y,1/2-z
	C(1)–H(1)···O(1)	1.08	2.41	3.212(2)	130	1/2-x,-1/2+y,1/2-z
**SA2(RT)**	N(1)–H(1N)···O(1)	1.01	1.55	2.556(3)	178	-1+x,1/2-y,1/2+z
	C(1)–H(1)···O(2)	1.08	2.42	3.197(4)	128	-1+x,1/2-y,1/2+z
**SA2(LT)**	N(1)–H(1N)···O(1)	1.01	1.54	2.545(2)	179	x,1/2-y,1/2+z
	C(1)–H(1)···O(2)	1.08	2.35	3.160(3)	131	x,1/2-y,1/2+z
**CO1(RT)**	O(1)–H(1A)···N(1)	0.98	1.65	2.629(3)	172	1+x,y,z
	C(1)–H(1)···O(2)	1.08	2.29	3.172(3)	137	-1+x,y,z
**CO1(LT)**	O(1)–H(1A)···N(1)	0.98	1.64	2.620(3)	173	x,y,1+z
	C(1)–H(1)···O(2)	1.08	2.30	3.144(3)	134	x,y,-1+z
**CNT1(RT)**	N(1)–H(1N)···O(1)	1.01	1.64	2.550(5)	148	
**CNT1(LT)**	N(1)–H(1N)···O(1)	1.01	1.52	2.525(4)	176	

Table S3 Hydrogen bond geometrical parameters (Bruker) of **CNT1**.

Compound	Interaction	D–H/ Å	H···A/ Å	D···A/ Å	∠D–H···A/ º	Symmetry
**CNT1(RT)**	O(1)–H(1)···N(1)	0.91(5)	1.65(5)	2.548(4)	170(5)	-1+x,y,z
**CNT1(LT)**	O(1)–H(1)···N(1)	1.13(5)	1.40(5)	2.523(4)	175(5)	

Table S4 Hydrogen bond geometrical parameters (Bruker) of **CNT1** (O–H, N–H and C–H distances are neutron normalized).

Compound	Interaction	D–H/ Å	H···A/ Å	D···A/ Å	∠D–H···A/ °	Symmetry
**CNT1(RT)**	O(1)–H(1)···N(1)	0.98	1.58	2.548(4)	169	-1+x,y,z
**CNT1(LT)**	N(1)–H(1)···O(1)	0.98	1.54	2.523(4)	175	

S5. ORTEP diagram for SA1, SA2, CO1, and CNT1 (Rigaku)

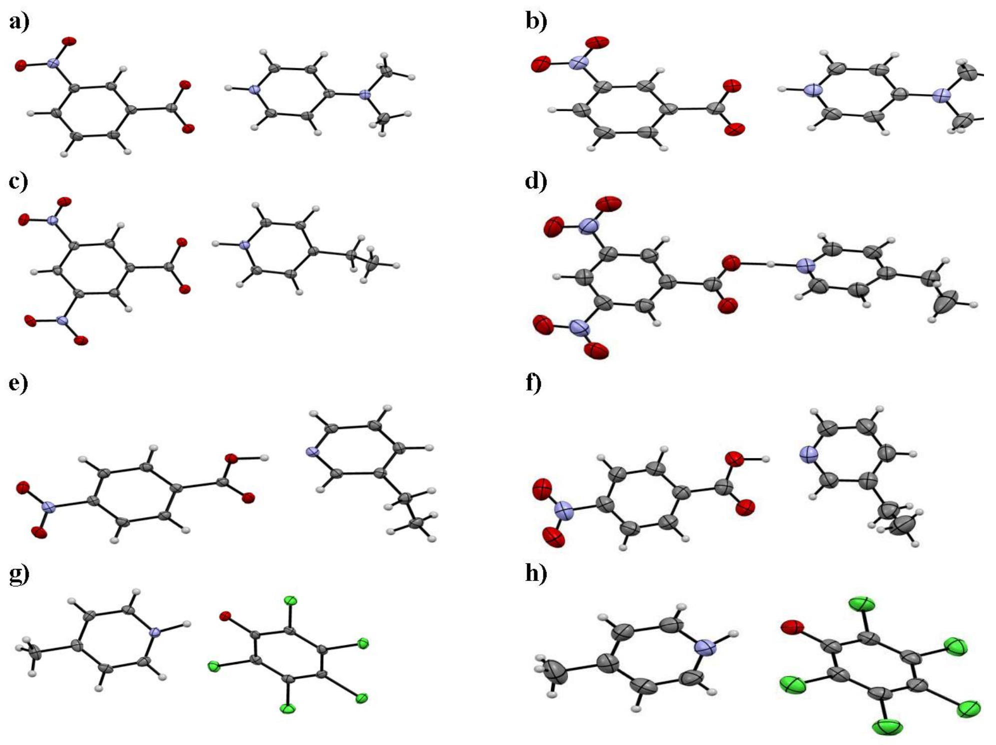

Figure S13 a) **SA1** at -163 °C, b) **SA1** at 25 °C, c) **SA2** at -163 °C, d) **SA2** at 25 °C, e) **CO1** at -163 °C, f) **CO1** at 25 °C, g) **CNT1** at -163 °C, h) **CNT1** at 25 °C.

S6. Calculated invCP-VC spectra of CO1

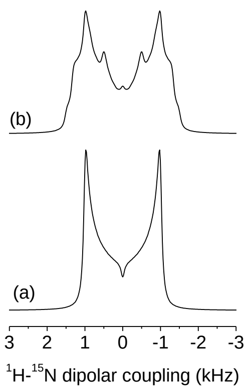

Figure S14 Calculated invCP-VC spectra of **CO1** (a) include only nearest ^{1}H-^{15}N interactions and (b) include three ^{1}H-^{15}N interactions.

S7. ssNMR 1D spectra of SA1, SA2, CO1, and CNT1

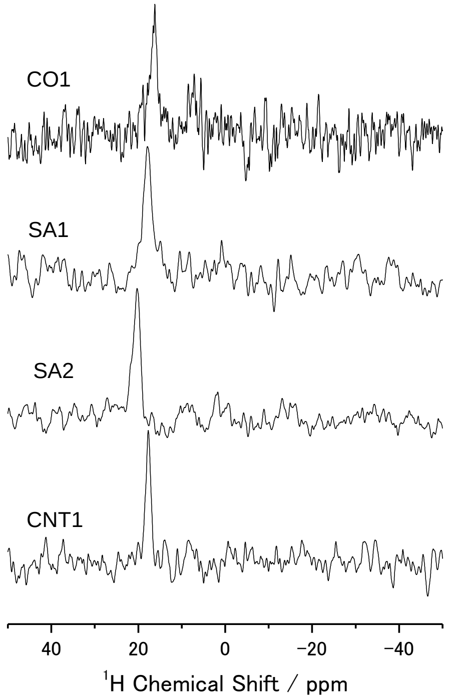

Figure S15 ^{1}H→^{15}N→^{1}H filtered 1H NMR spectra of **CO1**, **SA1**, **SA2**, and **CNT1**. The spectra are obtained by summing the CP-VC spectra with a contact time < 300 μs to remove the remote magnetization transfer.
